# Head Circumference Versus Length and Weight Deficits up to 2 Years of Age in Bangladesh

**DOI:** 10.1111/mcn.13793

**Published:** 2024-12-26

**Authors:** Leanna Cho, Alison S. B. Dasiewicz, Kelly M. Watson, Huma Qamar, Diego G. Bassani, Stanley Zlotkin, Minhazul Mohsin, Farhana Khanam Keya, Prakesh S. Shah, Davidson H. Hamer, Abdullah Al Mahmud, Daniel E. Roth

**Affiliations:** ^1^ Centre for Global Child Health, The Hospital for Sick Children Toronto Canada; ^2^ Department of Paediatrics University of Toronto Toronto Canada; ^3^ Department of Nutritional Sciences University of Toronto Toronto Canada; ^4^ Program in Public Health University of California Irvine California USA; ^5^ Nutrition and Clinical Services Division International Centre for Diarrhoeal Disease Research, Bangladesh (icddr,b) Dhaka Bangladesh; ^6^ Department of Pediatrics Mount Sinai Hospital Toronto Canada; ^7^ Department of Global Health Boston University School of Public Health Boston Massachusetts USA; ^8^ Section of Infectious Diseases, Boston University Chobanian & Avedisian School of Medicine Boston Massachusetts USA

**Keywords:** anthropometry, cephalometry, child development, crown‐rump length, developing countries, growth and development, malnutrition

## Abstract

Infant undernutrition, defined by length‐ and weight‐based indices, is common in low‐ and middle‐income countries (LMICs), but corresponding deficits in head size have received less attention. In a cohort of term newborns in Dhaka, Bangladesh, we compared the severity of deficits (vs. World Health Organization Growth Standards) in head circumference (HC), length and weight at birth and every 3 months until 2 years of age (*n* range across timepoints: 843–920). We estimated the mean and 25th, 50th and 75th percentiles of HC‐, length‐ and weight‐for‐age z‐scores (HCZ, LAZ and WAZ, respectively). Differences between HCZ and LAZ (or WAZ) were analyzed using paired *t* tests and quantile regression. We also derived HCZ using height‐age instead of chronological age at 3–24 months. Mean HCZ was significantly higher than mean LAZ and WAZ at birth, but HCZ was significantly lower than LAZ at 6, 9 and 12 months and the HCZ and LAZ deficits were similar from 15 to 24 months. Mean HCZ was lower than WAZ at all ages beyond birth. Patterns were broadly consistent at the 25th, 50th and 75th percentiles. The HCZ deficit remained evident when HC was standardized using height‐age at all ages beyond birth, indicating HC was reduced relative to body size. In conclusion, among term‐born children in Dhaka, HCs were smaller than international standards at all ages up to 2 years, and there was no evidence of postnatal head sparing. Consideration should be given to routine measurement of HC in population health surveys in LMICs.

## Summary

- In a Bangladeshi cohort of term infants, average head circumference (HC) z‐scores were closer to international norms than corresponding length and weight z‐scores at birth, suggesting possible foetal head sparing.
- Postnatal head sparing was not observed among children in Dhaka at any timepoint following birth. At 6, 9 and 12 months of age, the HC distribution was further below the international norm compared to the length and weight distributions. Analyses using height‐age rather than chronological age showed that postnatal HC deficits were more severe than expected given children's average height, at all timepoints following birth.
- The entire HC distribution was negatively displaced relative to the international standard at all ages, indicating that the head size deficit is a whole‐population condition, analogous to linear growth faltering.
- HC, like length and weight, should be considered for routine monitoring in LMIC settings where early childhood undernutrition is common.

## Graphical abstract

Term infants in Bangladesh had smaller head circumferences compared to international standards. In the postnatal period (3–24 months of age), average deficits in head circumference were more severe than expected based on body lengths, indicating a lack of head‐sparing in a setting where early childhood undernutrition is widespread.

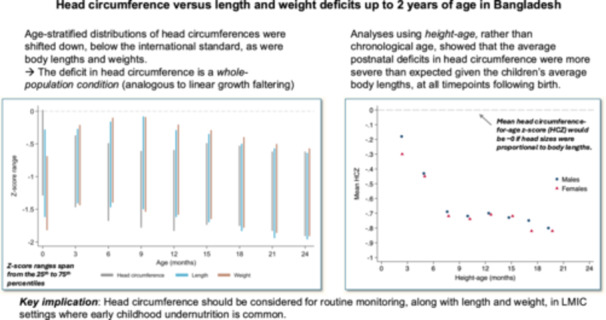

**Abbreviations**
CIs: confidence intervals
ERC: Ethical Review Committee
HAZ: height‐for‐age z‐score
HC: head circumference
HCZ: head circumference‐for‐age z‐score
LMICs: low and middle‐income countries
MDIG: maternal vitamin D for infant growth
REB: Research Ethics Board
SD: standard deviation
SickKids: The Hospital for Sick Children
SQ‐LNS: small‐quantity lipid‐based nutrient supplements
WAZ: weight‐for‐age z‐score
WHO‐GS: World Health Organization growth standards

## 1 Introduction

In young children, inadequate dietary intake and other adverse conditions contribute to suboptimal growth and development. Deficits in height growth and insufficient weight gain for age are the most commonly used indicators of undernutrition in low‐ and middle‐income countries (LMICs). Head circumference (HC) is widely measured in paediatric practice and research (WHO 2007) but infrequently measured in population‐based anthropometric surveys in LMICs, and small HC is not widely used as an indicator of child health in public health practice.

The rationale for measuring HC in young children is that cranial bone formation, which occurs via intramembranous ossification, is driven by an increase in brain size (Galea et al. 2021; Jin, Sim, and Kim 2016), such that HC reflects intracranial volume in infants and young children (Bartholomeusz, Courchesne and Karns 2002; Bray et al. 1969; Martini et al. 2018). Brain growth and cognitive function are impaired under adverse environmental conditions (Gewa et al. 2009; Miller et al. 2016; Stoch et al. 1982; Winick and Brasel 1977), and numerous studies have found a positive correlation between HC at birth and during early childhood with later cognitive ability (Gale et al. 2006; Heinonen et al. 2008; Koshy et al. 2021; Veena et al. 2010).

In contrast to cranial bones, long bone growth occurs through endochondral ossification, which requires a cartilage matrix before bone can be formed and is, therefore, regulated by distinct mechanisms (Galea et al. 2021). In generally healthy children, body mass and length have been found to follow different patterns than head growth (Scheffler, Greil, and Hermanussen 2017). *Head sparing*, whereby HC growth is relatively preserved compared to other anthropometric parameters, has been observed mainly in the context of intrauterine growth restriction and is thought to be mediated by preferential blood flow to the brain under conditions of nutrient inadequacy (Cohen, Baerts, and van Bel 2015). Head sparing has also been observed in high‐income settings when children experience a prolonged period of inadequate postnatal nutrition (Jaffe 2011; Larson‐Nath and Goday 2016; Tang et al. 2021).

The extent to which head sparing occurs throughout infancy in the context of widespread undernutrition and consequent linear growth faltering in LMICs is not well described. A study of undernourished children in rural Nepal showed no evidence of head sparing in the first 7 years of life, as mean HC‐for‐age z‐scores (HCZ) were lower than mean height‐for‐age z‐scores (HAZ) at all evaluated timepoints; however, this study did not measure children at birth (Miller et al. 2016). Lack of head sparing (HCZ vs*.* weight‐for‐age z‐score [WAZ]) was also found in Tanzanian Maasai newborns after mothers experienced calorie restriction during the third trimester, but measurements were limited to weight and HC at birth (Powell et al. 2020). A higher proportion of children had HCZ < −2 (i.e., more than 2 standard deviations below the reference population median of head size) than the prevalence of stunting (HAZ < −2) or wasting (weight‐for‐length z‐score [WLZ] < −2) at 1, 12 and 24 months of age in semi‐urban settlements of South India (Sindhu et al. 2019). These findings suggest that head size deficits compared to international norms may be more severe than those related to length and weight in LMICs; however, few studies have examined the relationship of head size with length or weight at birth and, thereafter, or established evidence for or against postnatal head sparing in resource‐limited settings.

The aim of this study was to explore whether there is relative preservation of HC compared to body size indices in a cohort of infants in Dhaka, Bangladesh. Specifically, we compared standardized HC measurements, based on the WHO Growth Standards (WHO‐GS), to corresponding standardized length or weight in discrete age windows from birth to 2 years of age.

## 2 Methods

### 2.1 Data Source

This was a secondary analysis of data from the Maternal Vitamin D for Infant Growth (MDIG) trial, a double‐blind, placebo‐controlled, dose‐ranging clinical trial evaluating the effect of prenatal and postpartum vitamin D supplementation on infant linear growth in Dhaka, Bangladesh (clinicaltrials.gov registration: NCT01924013) (Roth et al. 2018). Ethics approval for the MDIG trial was obtained from the Hospital for Sick Children (SickKids) Research Ethics Board (REB#1000039072) and the International Centre for Diarrhoeal Disease Research, Bangladesh Ethical Review Committee (ERC PR number 13055). Secondary use of MDIG trial data in this study was approved by the SickKids REB (REB#1000080094).

In the MDIG trial, visits were scheduled weekly until 6 months postpartum, after which visits were scheduled every 3 months until 24 months postpartum. Data collected from mothers and children included anthropometry, demographics and household conditions. Infant length, weight and HC were measured at birth, at one randomly assigned weekly visit during the first 2 months of life, and then every 3 months starting at the 3‐month visit, using procedures adapted from the INTERGROWTH‐21st study (Cheikh Ismail et al. 2013; Roth et al. 2015).

Infants from the MDIG trial were included in this study based on the following criteria: (i) born at or after 37 gestational weeks, (ii) HC, length and weight obtained within 7 days of at least one of the following target ages: 0, 3, 6, 9, 12, 15, 18, 21 and 24 months (i.e., 15‐day window, except for *birth* which allowed for an 8‐day window), (iii) no major congenital anomalies noted at birth. As maternal vitamin D supplementation did not affect child length, weight or HC in the original trial analyses (Roth et al. 2018), infants from all the trial intervention groups were included in this study. If multiple measurements were obtained within a 15‐ (or 8‐) day window, the set of HCZ, LAZ and WAZ closest to the target age was used. If HCZ, LAZ and WAZ were not all measured on a single day within a 15‐ (or 8‐) day window but were all obtained within the same window, the HCZ, LAZ and WAZ measured on different days were used to create one set of measurements.

### 2.2 Outcome Measures

We estimated the mean, and 25th, 50th and 75th percentiles of HCZ, LAZ and WAZ, generated according to the WHO‐GS based on chronological age at designated target ages (0, 3, 6, 9, 12, 15, 18, 21 and 24 months), and overall and by sex. We assumed that the z‐score distributions for the three parameters based on the WHO‐GS could be reasonably compared to one another, cross‐sectionally at a given age (e.g., mean HCZ vs*.* mean LAZ at age 12 months), as these metrics are standardized using the same methods and reference population from the WHO Multicenter Growth Reference Study (WHO 2006, 2007; WHO Multicentre Growth Reference Study Group 2006).

Secondary outcomes were the proportions of children with z‐scores less than −2 (relative to the WHO‐GS median) for HCZ, LAZ and WAZ, at each age timepoint. Proportions of children with z‐scores below −3 are not reported as they were too low to enable meaningful comparisons between the anthropometric parameters.

### 2.3 Statistical Analyses

Potential outliers were identified by visual inspection and by using the WHO convention of flagging implausible z‐scores (z‐score less than −6 or greater than +6 for LAZ and WAZ; ±5 for HCZ) (WHO 2007.). Maternal‐infant characteristics of the final study sample included in this analysis are summarized using descriptive statistics. Outcome measures were estimated with 95% confidence intervals (95% CI) for each target age (0, 3, 6, 9, 12, 15, 18, 21 and 24 months), overall, and by sex. Primary outcomes were visualized using scatterplots and range plots, and the statistical significance of the mean differences between HCZ and LAZ or HCZ and WAZ within each age or age‐sex stratum was tested using paired *t* tests and quantile regression (for differences between 25th, 50th and 75th percentiles).

Analyses were designed to quantify and compare population‐average deficits in HC, length and weight in relation to international normative standards (the WHO‐GS), primarily by examining the magnitudes of the negative displacements of the z‐score distributions. We assumed that an HCZ distribution that is negatively displaced to a similar or lower location than the age‐matched LAZ or WAZ distributions would provide evidence against head sparing at the given age timepoint. Although a relatively higher HCZ (i.e., closer to the WHO‐GS median than length or weight) may be consistent with head sparing, the minimum absolute difference between mean HCZ and LAZ (or WAZ) that would support head sparing is age‐dependent due to differences in the shape of the median HC and length (and weight) trajectories (WHO 2006, 2007). We, therefore, conducted a supplementary analysis using height‐age to assess the proportionality of HC given the observed average length (see below).

### 2.4 Supplementary Analyses

Correlations between corresponding (i.e., age/sex stratum‐matched) mean HCZ and mean LAZ, and between mean HCZ and mean WAZ, were estimated for two age ranges (0 to 12 months inclusive; 15–24 months inclusive) using Pearson and Spearman correlation coefficients. We did not estimate correlations between anthropometric parameters at the individual child level because we were not interested in whether shorter/lighter children had smaller head sizes relative to peers, but rather the extent to which negative displacement of the population distribution of HC corresponded to shifts of the length or weight distributions.

We also stratified primary outcomes by mode of delivery, such that differences in mean HCZ, mean LAZ and mean WAZ were compared for infants delivered by C‐section or vaginally. Results disaggregated by mode of delivery were visualized at each target age using scatterplots. In a separate supplementary analysis, mean HCZ was re‐expressed using mean height age instead of chronological age at timepoints from 3 to 24 months (chronological). Height age was defined as the age at which the observed mean length of children was equal to the WHO‐GS median. Mean length was, therefore, calculated at each target age, disaggregated by sex and compared to the WHO‐GS median lengths (available in the ‘lenanthro.dta’ file) (WHO 2007.); the age corresponding to the median length that was closest to the observed mean length was selected as the height‐age. We did not calculate height‐age at birth as the mean birth length in the cohort was below the WHO‐GS median length at birth, and methods have not yet been developed to determine height‐age in this circumstance. Mansukoski et al. provide additional details and sample Stata code to calculate height‐age (Mansukoski et al. 2022).

## 3 Results

**Figure 1:**
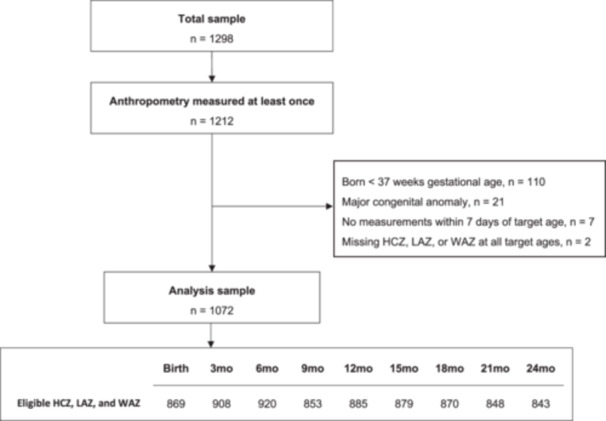
Participant selection flow chart. HCZ, head circumference‐for‐age z‐score; LAZ, length‐for‐age z‐score; WAZ, weight‐for‐age z‐score.

Among 1212 infants with at least one HC measurement between birth and 24 months of age, 140 (13%) were excluded from this analysis due to preterm birth (*n *= 110), major congenital anomalies (*n *= 21), not having an HCZ within 7 days of any of the target ages (*n *= 7), or missing LAZ, WAZ or HCZ across all target ages (such that the required triad was not available at any of the target ages) (*n *= 2). Of the remaining 1072 eligible participants, final sample sizes at each target age ranged from 843 to 920 after excluding participants measured more than 7 days from each target age (Figure 1). The baseline characteristics of the included participants are shown in Table 1.

**Table 1:** Baseline characteristics.

Characteristics	
*N*	1072
Male sex, *n* (%)	543 (51)
Vaginal birth, *n* (%)^a^	398 (45.8)
HCZ, mean ± SD^b^	−1.28 ± 0.89
HCZ < −2, *n* (%)^b^	176 (21)
LAZ, mean ± SD^b^	−1.31 ± 1.05
LAZ < −2, *n* (%)^b^	204 (24)
WAZ, mean ± SD^b^	−1.19 ± 1.14
WAZ < −2, *n* (%)^b^	189 (22)
Quintiles of Household asset index, *n* (%)^c^	
1 (lowest)	219 (21)
2	201 (19)
3	218 (20)
4	208 (20)
5 (highest)	218 (20)
Maternal characteristics^d^	
Age (years), median (range)	22 (18, 39)
Height (cm), mean ± SD	151 ± 5
Gravidity, median (range)^e^	2 (1, 9)
Completed secondary education or more, *n* (%)	235 (22)
Maternal vitamin D supplementation group^f^, *n* (%)	
0 IU/week: 0 IU/week	209 (19)
2400 IU/week: 0 IU/week	222 (21)
1,6800 IU/week: 0 IU/week	213 (20)
2,8000 IU/week: 0 IU/week	211 (20)
2,8000 IU/week: 2,8000 IU/week	217 (20)

^a^ Measured at target age of 0 months (*n* = 869).

^b^ Measured at target age of 24 months (*n* = 843).

^c^ Measured from overall data set with some missingness (*n* = 1064).

^d^ Collected at 7−24 weeks of gestation.

^e^ Gravidity defined as number of pregnancies including current pregnancy.

^f^ Vitamin D supplementation denoted as prenatal: postpartum.

**Figure 2:**
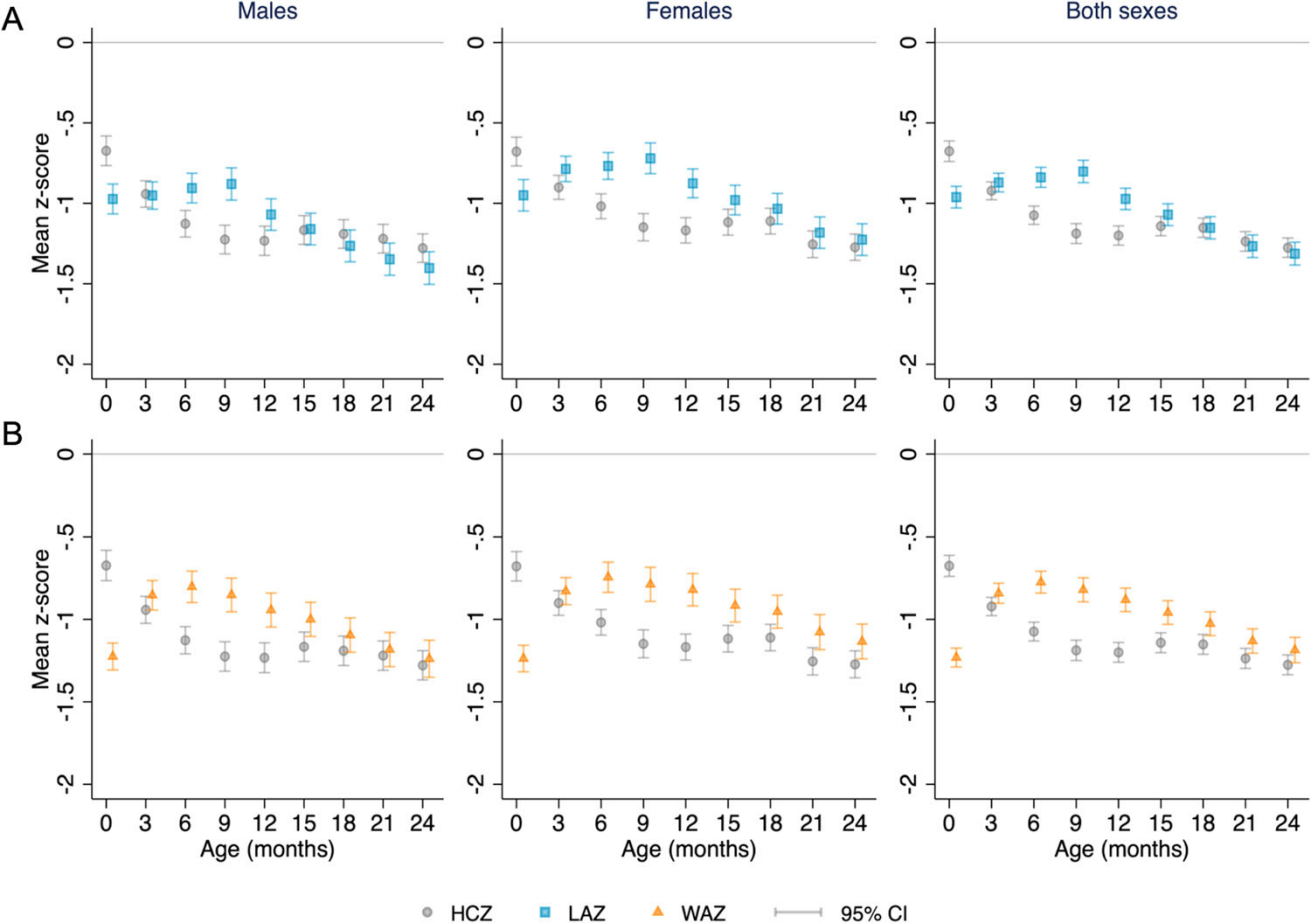
Mean head circumference‐for‐age z‐score (HCZ) compared to mean length‐for‐age z‐score (LAZ) (A), and mean HCZ compared to mean weight‐for‐age z‐score (WAZ) (B) at tri‐monthly measurements from birth to 24 months, for all children, and by sex.

**Figure 3:**
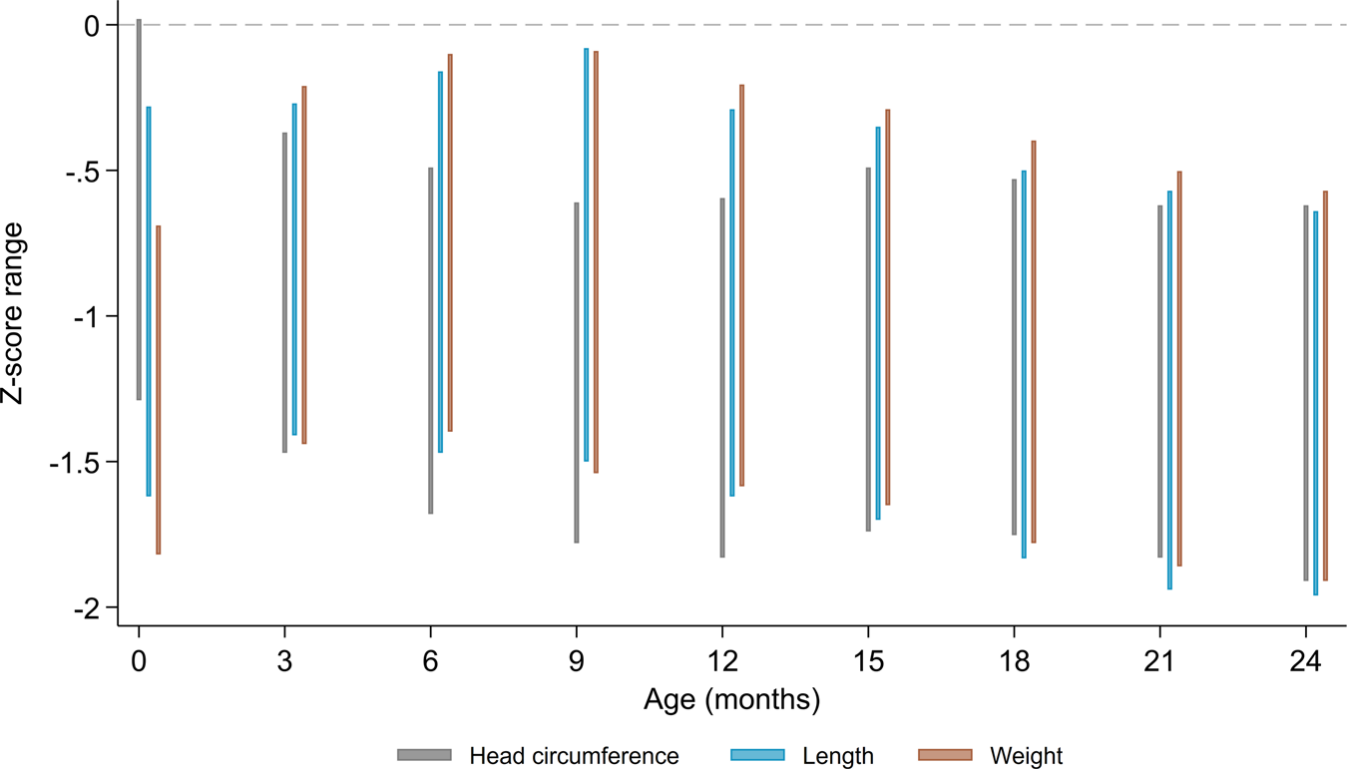
Range plot of the 25th and 75th percentiles of head circumference‐for‐age z‐scores (HCZ), length‐for‐age z‐scores (LAZ) and weight‐for‐age z‐scores (WAZ), sexes combined.

At birth, mean HCZ was significantly higher than mean LAZ (MD [95% CI]: +0.29 [0.22, 0.35]) and mean WAZ (+0.56 [0.51, 0.60]) (Figure 2 and Supporting Information S1: Table 1). At 3 months of age, mean HCZ was not significantly different from mean LAZ. At 6, 9 and 12 months, mean HCZ was significantly lower than mean LAZ, and at all timepoints from 15 to 24 months, mean HCZ and mean LAZ were similar (Figure 2A and Supporting Information S1: Table 1). Mean HCZ was significantly lower than the corresponding mean WAZ at all timepoints between 3 and 24 months of age (Figure 2B and Supporting Information S1: Table 1). Patterns were similar in analyses disaggregated by sex (Figure 2 and Supporting Information S1: Table 1). Deficits in HCZ, LAZ and WAZ were due to negative displacements of the entire z‐score distributions (Figure 3), such that HCZ‐LAZ (or HCZ‐WAZ) differences were generally similar at the 25th, 50th and 75th percentiles of HCZ, LAZ and WAZ at each timepoint (Supporting Information S1: Table 2).

Compared to the proportions of children with LAZ < −2 or WAZ < −2, the proportions of children with HCZ < −2 were lower at birth, slightly higher at 6, 9 and 12 months of age, and about the same at timepoints from 15 to 24 months of age (Supporting Information S1: Table 3). Weaker correlations were observed between mean HCZ and either mean LAZ or mean WAZ between birth and 12 months compared to the correlations based on encounters between 15 and 24 months of age (Supporting Information S1: Figure 1).

When participants were stratified by mode of delivery, mean HCZ was higher at birth for babies delivered by C‐section versus vaginally; however, groups of infants delivered either vaginally or by C‐section had higher mean HCZ than mean LAZ and mean WAZ at birth (Supporting Information S1: Figure 2).

**Figure 4:**
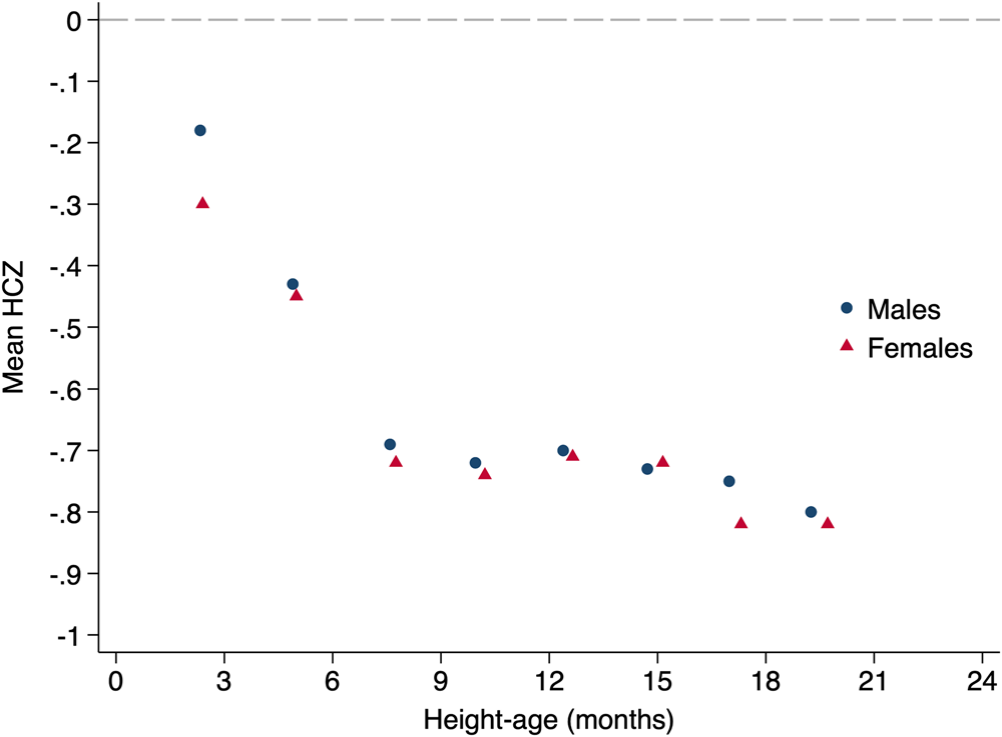
Head circumference‐for‐age z‐score (HCZ) calculated using height‐age instead of chronological age for males and females.

When mean HCZ was re‐expressed using mean height‐age, mean HCZ values from 3 to 24 months were always closer to 0 than corresponding HCZ values based on chronological age; however, the HCZ deficit remained evident at all ages and was most pronounced at older ages (Figure 4; see Figure 2A for corresponding HCZs based on chronological age).

## 4 Discussion

Among term‐born children in a setting where foetal and postnatal linear growth faltering is pervasive, average age‐ and sex‐standardized HC was greater than the corresponding standardized measures of average body length (LAZ) or weight (WAZ) only at birth, whereas at older ages (3 months to 24 months), deficits in HC relative to the WHO‐GS were similar to or larger than those observed for length or weight. Therefore, despite possible foetal head sparing based on birth anthropometry, there was no head sparing at any later postnatal age. On the contrary, we found evidence that postnatal deficits in head size (at 3 months and beyond) were more severe than expected given the children's observed body sizes. The entire HC distribution was negatively displaced relative to international standards, which implies that children with head sizes above and below the population average had smaller HCs than expected for their chronological age based on international standards; this pattern indicates that the head size deficit is a whole‐population condition, similar to linear growth faltering (Roth et al. 2017).

Our findings were generally consistent with previous studies in LMICs that showed deficits in HC for age relative to international norms (Miller et al. 2016; Nicolaou et al. 2020; Sindhu et al. 2019; Stoch et al. 1982). At birth, we found that while average head size was below the median of the international normative (WHO‐GS) head size distribution, it was nonetheless closer to the WHO‐GS median than were average LAZ and average WAZ, suggesting the possibility of relative head sparing in utero. This was consistent with an observational study of foetal growth in a Bangladeshi cohort in which mean HC was found to be greater than the WHO median at 37 weeks gestational age, whereas abdominal circumference and femur length were below the WHO reference (Ferdous et al. 2018). We considered whether the high percentage of C‐section deliveries in the present study population (> 50%), which was almost double that in the WHO‐GS reference population (27%) (WHO Multicentre Growth Reference Study Group 2006), could have partly accounted for the relatively higher HC z‐scores observed at birth compared to later ages. Infants born by C‐section tend to experience less pronounced or no head moulding during delivery (Bronfin 2001), which could result in rounder heads with slightly larger occipital‐frontal diameters. While we did find that the mean HCZ was higher for infants born via C‐section versus vaginally, the mean HCZ remained higher than mean LAZ and mean WAZ at birth in both groups, indicating that this pattern was not explained by mode of delivery.

The observation that the HC and length deficits on the z‐score scales were similar at timepoints from 15 to 24 months could be interpreted as suggesting that head and body sizes became proportional by 2 years. However, the analysis of head size in relation to height‐age challenged this conclusion by showing that head sizes were not only below international norms at all chronological ages, but also smaller than would be expected for healthy children of the same lengths. Height‐age is the age at which the observed length of children equals the WHO‐GS median length and, therefore, when LAZ (for height‐age) is 0. If HCZ deficits relative to the WHO‐GS median were of equivalent magnitude to height‐age, HCZ would be approximately 0 at all target ages. The persistence of HCZ < 0 at each target age when normalized to height‐age indicated that the postnatal HCZ deficit (relative to the WHO‐GS) is worse than, rather than proportional to, the length deficit.

These findings suggest head growth after birth may be more sensitive to growth‐inhibiting factors than height or weight, or is influenced by different factors than those affecting long bone growth and fat mass accumulation. Nutrition is a key factor that influences growth, but while suboptimal linear growth and weight gain are widely recognized as primary features of undernutrition in LMICs, small HC is generally only regarded as a late sign of severe malnutrition (Tang et al. 2021). The relative effect of nutrition interventions on HC (compared to length and weight) is unclear, with some evidence suggesting that HC is less responsive to nutrition interventions in the prenatal period (Gresham et al. 2014; Lassi et al. 2021; Stevens et al. 2015). However, small‐quantity lipid‐based nutrient supplements (SQ‐LNS) provided to infants 6–24 months of age were found to significantly reduce the risk of small head size, in addition to effects on the prevalence of stunting and underweight (Dewey et al. 2021). SQ‐LNS may increase HC by ensuring the recommended nutrient intake for essential fatty acids in infants and young children (Arimond et al. 2015), which are vital for early brain development and positively associated with HC (Brockway et al. 2024; Xiang et al. 2000). Nutrition, therefore, may be a common determinant of length, weight and head size, with head size particularly sensitive to specific nutrients and/or timing of intervention.

Nonnutritional factors that disproportionately affect brain growth could also explain why the postnatal deficit in HC is more severe than for length and weight. For example, low HCZ has been associated with exposure to heavy metals, including cadmium (Kippler et al. 2012; Lin et al. 2011), arsenic (Rahman et al. 2008; Wai et al. 2020) and lead (Rothenberg et al. 1999; Schell et al. 2009; Zeng et al. 2019). In the present study population, we previously reported that exposure to heavy metals, particularly lead, was high, with 90% of maternal samples and 82% of cord blood samples exceeding acceptable exposure reference values (Jukic et al. 2020). The impact of heavy metal exposure is likely to be most significant during early ages when the brain is undergoing rapid growth and is particularly sensitive to chemical toxicity (Grandjean and Landrigan 2006, 2014).

A key strength of this study was the use of a high‐quality longitudinal anthropometric data set whereby each measurement was obtained by two independently trained personnel following standardized procedures with high interrater reliability (Roth et al. 2018). Although the WHO‐GS are the most widely used child growth standards worldwide (de Onis et al. 2012), a limitation of this study is the assumption that the WHO‐GS for head size, length and weight are all similarly suitable growth references for Bangladeshi children at all observed ages. Infants enroled in the India sites of the WHO and INTERGROWTH‐21st growth reference studies had, on average, lower birth weight, length and smaller HC compared to other sites in other countries (Villar et al. 2014; WHO Multicentre Growth Reference Study Group 2006), suggesting that the use of the WHO‐GS in South Asia may variably over‐estimate deficits of HCZ, LAZ and WAZ. Conversely, average HCs in many countries tend to be larger than the WHO‐GS HC median and between‐country variation in head size appears greater than for height or weight (Natale and Rajagopalan 2014), so using the WHO‐GS to generate HCZs in this study could have underestimated the severity of deficits in head size relative to the other anthropometric parameters. We also recognized that while analyses of z‐score distributions (HCZ vs. LAZ or WAZ) at discrete age timepoints enabled cross‐sectional comparisons of the indices to their respective international norms, the supplemental analyses in which HCZ was derived using height‐age instead of chronological age were ultimately preferred for inferences about head sparing because they accounted for the differences between the rates of change in the WHO‐GS median HC for age trajectory compared to the median length or weight trajectories (WHO 2006, 2007); however, since we only used a single set of growth standards (the WHO‐GS), which are truncated at 0 days, we did not generate height‐age at birth, and, therefore, remained uncertain about whether the differences in z‐scores at birth were evidence of foetal head sparing.

In summary, these findings combined with similar prior observations in other LMICs suggest that postnatal head sparing does not occur among term‐born children in settings where early childhood undernutrition is long‐standing and widespread. Despite possible in utero head sparing, postnatal deficits in HC relative to international norms were at least as severe as corresponding weight or length deficits, and head sizes were consistently smaller than would be expected for healthy children of the same heights. These findings suggest that greater attention should be focused on HC as a marker of brain growth in vulnerable populations, and highlight the potential value of incorporating routine measurements of HC in addition to length and weight parameters in health and nutrition surveys of children living in LMICs.

## Author Contributions

Leanna Cho, Alison S. B. Dasiewicz and Daniel E. Roth conceptualized the study; Leanna Cho, Alison S. B. Dasiewicz, Huma Qamar, Diego G. Bassani, Stanley Zlotkin, Prakesh S. Shah, Davidson H. Hamer and Daniel E. Roth designed the study; Minhazul Mohsin, Farhana Khanam Keya and Abdullah Al Mahmud supervised the collection of data; Alison S. B. Dasiewicz and Kelly M. Watson analyzed the data; Huma Qamar verified the statistical code; Leanna Cho, Kelly M. Watson and Daniel E. Roth wrote the paper. All authors reviewed, edited and approved the final version of the manuscript.

## Conflicts of Interest

The authors declare no conflicts of interest.

## Supporting information

Supporting information.

## Data Availability

Data described in the manuscript, code book and analytical code will be made available upon reasonable request to the authors. Transfer of deidentified individual participant data may require approval by a research ethics board, and data requestors may be required to sign a data access agreement.
