# Supplementary material for: Head Circumference Versus Length and Weight Deficits up to 2 Years of Age in Bangladesh
Source: Matern Child Nutr. 2024 Dec 26;21(2):e13793. doi: 10.1111/mcn.13793 (PMC11956067; doi:10.1111/mcn.13793)
Supplement: Supplementary file 1 — Supporting information. [file MCN-21-e13793-s001.docx]

**Supplementary Material**

**Head circumference versus length and weight deficits up to 2 years of age in Bangladesh**

**Supplemental Table 1** Differences (and 95% confidence intervals) between mean head circumference-for-age z-scores (HCZ) and either mean length-for-age z-scores (LAZ) or mean weight-for-age z-scores (WAZ), by age, from birth to 24 months of age in Bangladeshi infants.

| **Age (months)** | **N, overall** | **Difference (95% CI)** | | | | | |
| --- | --- | --- | --- | --- | --- | --- | --- |
|  |  | **HCZ-vs-LAZ** | | | **HCZ-vs-WAZ** | | |
|  |  | **Males** | **Females** | **Overall** | **Males** | **Females** | **Overall** |
| 0​ | 869 | **0.30 (0.21, 0.39)** | **0.27 (0.18, 0.37)** | **0.29 (0.22, 0.35)​** | **0.55 (0.48, 0.62)** | **0.56 (0.49, 0.63)** | **0.56 (0.51, 0.60)​** |
| 3​ | 908 | 0.01 (-0.08, 0.10) | **-0.11 (-0.19, -0.04)** | -0.05 (-0.11, 0.01)​ | **-0.09 (-0.17, -0.01)** | -0.07 (-0.15, 0.00) | **-0.08 (-0.13, -0.03)​** |
| 6​ | 920 | **-0.22 (-0.32, -0.13)** | **-0.25 (-0.34, -0.16)** | **-0.24 (-0.30, -0.17)​** | **-0.32 (-0.41, -0.24)** | **-0.27 (-0.35, -0.19)** | **-0.30 (-0.36, -0.24)​** |
| 9​ | 853 | **-0.35 (-0.45, -0.24)** | **-0.43 (-0.52, -0.33)** | **-0.39 (-0.46, -0.32)​** | **-0.37 (-0.46, -0.28)** | **-0.36 (-0.45, -0.27)** | **-0.37 (-0.43, -0.30)​** |
| 12​ | 885 | **-0.16 (-0.26, -0.06)** | **-0.29 (-0.38, -0.20)** | **-0.23 (-0.30, -0.16)​** | **-0.29 (-0.38, -0.19)** | **-0.35 (-0.44, -0.26)** | **-0.32 (-0.38, -0.25)​** |
| 15​ | 879 | -0.01 (-0.11, 0.10) | **-0.14 (-0.23, -0.04)** | -0.07 (-0.14, 0.00)​ | **-0.17 (-0.26, -0.07)** | **-0.20 (-0.29, -0.11)** | **-0.18 (-0.25, -0.12)​** |
| 18​ | 870 | 0.07 (-0.03, 0.18) | -0.08 (-0.17, 0.02) | 0.00 (-0.07, 0.07)​ | -0.10 (-0.19, 0.00) | **-0.16 (-0.25, -0.07)** | **-0.13 (-0.19, -0.06)​** |
| 21​ | 848 | 0.13 (0.02, 0.23) | -0.07 (-0.17, 0.02) | 0.03 (-0.04, 0.10)​ | -0.04 (-0.13, 0.06) | **-0.18 (-0.27, -0.09)** | **-0.11 (-0.17, -0.04)​** |
| 24​ | 843 | 0.12 (0.02, 0.23) | -0.05 (-0.14, 0.05) | 0.04 (-0.03, 0.11)​ | -0.04 (-0.14, 0.06) | **-0.14 (-0.23, -0.05)** | **-0.09 (-0.16, -0.02)​** |

Estimates are in bold if the 95% CI excluded the null. 95% CI, 95% confidence interval; HCZ, head circumference-for-age z-score; LAZ, length-for-age z-score; WAZ, weight-for-age z-score.

**Supplemental Table 2** Differences (and 95% confidence intervals) between the 25^th^, 50^th^, and 75^th^ percentiles of the head circumference-for-age z-score (HCZ) and the length-for-age z-score (LAZ) distribution, or the HCZ and the weight-for-age z-score (WAZ) distribution, by age, from birth to 24 months of age in Bangladeshi infants.

| **Age (months)** | **N, overall** | **Difference (95% CI)** | | | | | |
| --- | --- | --- | --- | --- | --- | --- | --- |
|  |  | **HCZ-vs-LAZ** | | | **HCZ-vs-WAZ** | | |
|  |  | **25^th^ Percentile** | **50^th^ Percentile** | **75^th^ Percentile** | **25^th^ Percentile** | **50^th^ Percentile** | **75^th^ Percentile** |
| 0​ | 869 | **0.33 (0.44, 0.22)​** | **0.29 (0.39, 0.20)** | **0.30 (0.41, 0.19)​** | **0.26 (0.31, 0.22)** | **0.29 (0.33, 0.24)** | **0.35 (0.40, 0.31)​** |
| 3​ | 908 | -0.06 (0.04, -0.16)​ | -0.07 (0.01, -0.15) | **-0.10 (-0.01, -0.19)​** | -0.01 (0.03, -0.06) | **-0.05 (-0.01, -0.10)** | **-0.08 (-0.04, -0.12)​** |
| 6​ | 920 | **-0.21 (-0.12, -0.30)​** | **-0.20 (-0.11, -0.29)** | **-0.33 (-0.23, -0.43)​** | **-0.14 (-0.09, -0.20)** | **-0.16 (-0.11, -0.21)** | **-0.19 (-0.15, -0.24)​** |
| 9​ | 853 | **-0.28 (-0.17, -0.39)​** | **-0.41 (-0.32, -0.50)** | **-0.53 (-0.42, -0.64)​** | **-0.12 (-0.07, -0.17)** | **-0.19 (-0.15, -0.23)** | **-0.26 (-0.21, -0.31)​** |
| 12​ | 885 | **-0.21 (-0.10, -0.32)​** | **-0.24 (-0.14, -0.34)** | **-0.31 (-0.20, -0.42)​** | **-0.12 (-0.07, -0.18)** | **-0.17 (-0.12, -0.21)** | **-0.19 (-0.14, -0.25)​** |
| 15​ | 879 | -0.04 (0.06, -0.14)​ | -0.08 (0.02, -0.18) | **-0.14 (-0.02, -0.26)​** | -0.05 (0.00, -0.09) | **-0.12 (-0.08, -0.16)** | **-0.10 (-0.05, -0.15)​** |
| 18​ | 870 | 0.08 (0.18, -0.02)​ | -0.03 (0.06, -0.12) | -0.03 (0.08, -0.14)​ | 0.01 (0.07, -0.04) | **-0.06 (-0.02, -0.11)** | **-0.06 (-0.01, -0.12)​** |
| 21​ | 848 | **0.11 (0.21, 0.01)​** | 0.02 (0.12, -0.08) | -0.05 (0.07, -0.17)​ | 0.01 (0.06, -0.03) | **-0.06 (-0.01, -0.11)** | -0.06 (0.00, -0.12)​ |
| 24​ | 843 | 0.05 (0.15, -0.05)​ | 0.04 (0.14, -0.06) | 0.02 (0.13, -0.09)​ | 0.00 (0.05, -0.05) | -0.04 (0.01, -0.08) | -0.03 (0.03, -0.08)​ |

Estimates are in bold if the 95% CI excluded the null. 95% CI, 95% confidence interval; HCZ, head circumference-for-age z-score; LAZ, length-for-age z-score; WAZ, weight-for-age z-score.

**Supplemental Table 3** Prevalence of head circumference-for-age z-scores (HCZ) <-2, length-for-age z-scores (LAZ) <-2, and weight-for-age z-scores (WAZ) <-2, by age, from birth to 24 months of age in Bangladeshi infants.

| **Age**  **(months)**​ | **N,**  **overall** | **% with z-score <-2​ (95% CI)** | | |
| --- | --- | --- | --- | --- |
|  |  | **HCZ<-2** | **LAZ<-2** | **WAZ<-2** |
| 0​ | 869 | 8.4 (6.6, 10.2) | 15.0 (12.6, 17.3) | 17.8 (15.3, 20.4) |
| 3​ | 908 | 9.4 (7.5, 11.3) | 10.5 (8.5, 12.5) | 10.6 (8.6, 12.6) |
| 6​ | 920 | 13.4 (11.2, 15.6) | 11.5 (9.5, 13.6) | 12.2 (10.1, 14.3) |
| 9​ | 853 | 18.5 (15.9, 21.1) | 11.4 (9.2, 13.5) | 13.5 (11.2, 15.8) |
| 12​ | 885 | 19.0 (16.4, 21.6) | 14.7 (12.4, 17.0) | 14.9 (12.6, 17.3) |
| 15​ | 879 | 17.0 (14.5, 19.4) | 18.1 (15.5, 20.6) | 16.0 (13.6, 18.5) |
| 18​ | 870 | 16.1 (13.6, 18.5) | 20.2 (17.6, 22.9) | 17.1 (14.6, 19.6) |
| 21​ | 848 | 18.3 (15.7, 20.9) | 22.6 (19.8, 25.5) | 20.9 (18.1, 23.6) |
| 24​ | 843 | 20.9 (18.1, 23.6) | 24.2 (21.3, 27.1) | 22.4 (19.6, 25.2) |

95% CI, 95% confidence interval; HCZ, head circumference-for-age z-score; LAZ, length-for-age z-score; WAZ, weight-for-age z-score


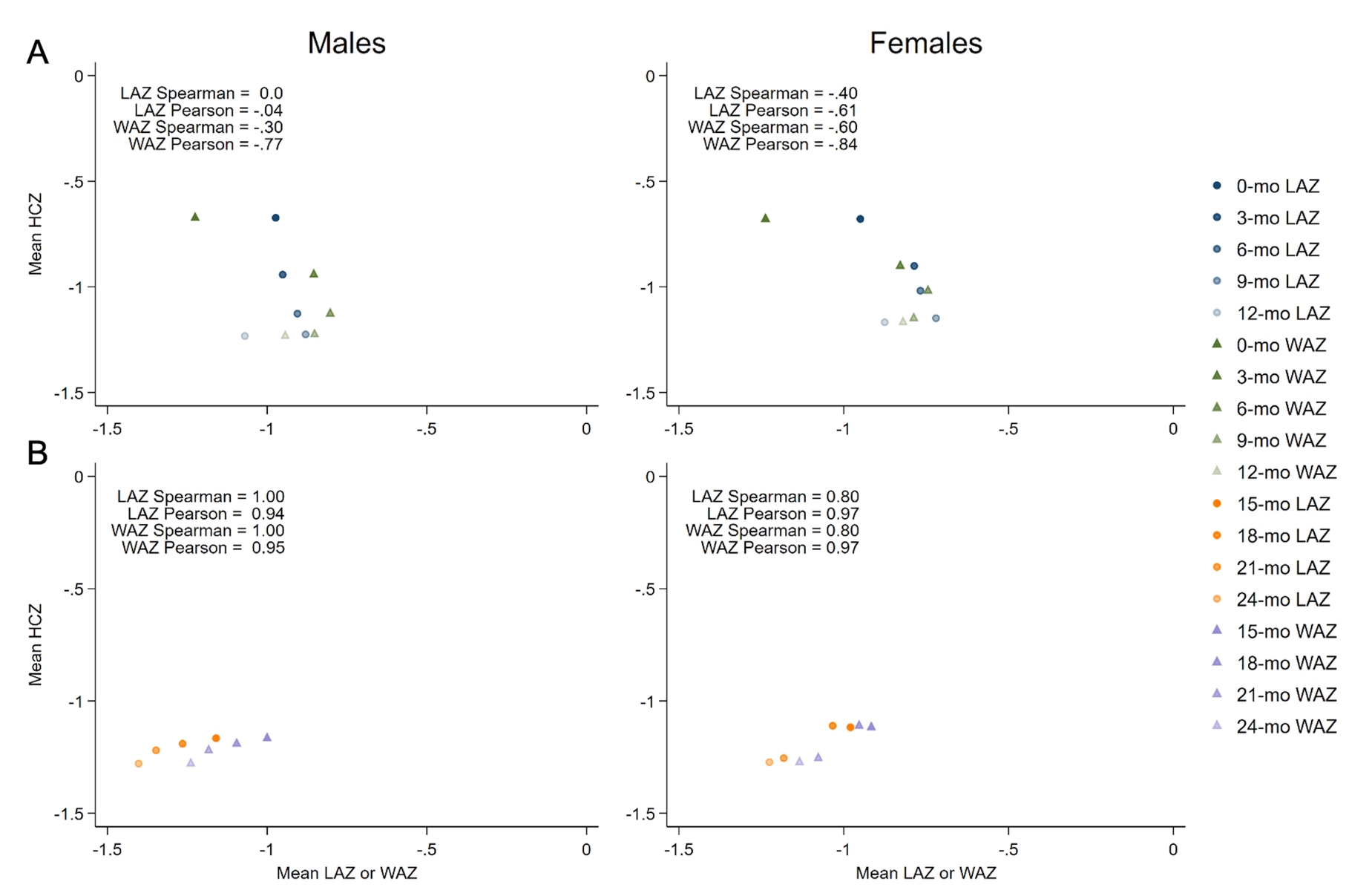


**Supplemental Figure 1** Relationship between mean HCZ and mean LAZ, or between mean HCZ and mean WAZ, from birth to 12 months (Panel A) and 15 to 24 months (Panel B) by sex. HCZ, head circumference-for-age z-score; LAZ, length-for-age z-score; WAZ, weight-for-age z-score.

**
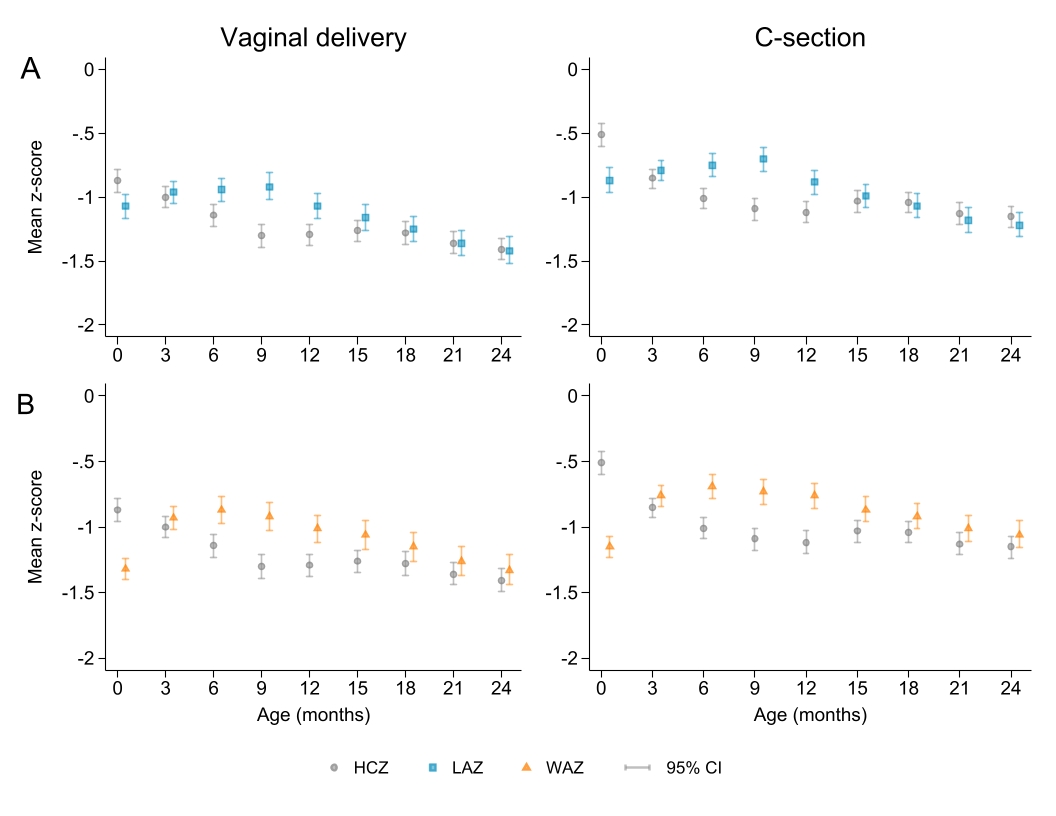
**

**Supplemental Figure 2** Comparison of mean HCZ versus mean LAZ (Panel A) and mean HCZ versus mean WAZ (Panel B) at tri-monthly measurements from birth to 24-months, by mode of delivery​. HCZ, head circumference-for-age z-score; LAZ, length-for-age z-score; WAZ, weight-for-age z-score.
